# The Effect of Curcumin and Cotrimoxazole in *Salmonella* Typhimurium Infection In Vivo

**DOI:** 10.1155/2013/601076

**Published:** 2013-08-29

**Authors:** Siwipeni Irmawanti Rahayu, Nurdiana Nurdiana, Sanarto Santoso

**Affiliations:** ^1^Laboratory of Microbiology, Faculty of Medicine, Brawijaya University, Jl Veteran Malang 65145, East Java, Indonesia; ^2^Laboratory of Pharmacology, Faculty of Medicine, Brawijaya University, Jl Veteran Malang 65145, East Java, Indonesia

## Abstract

Typhoid fever is a disease caused by *Salmonella* Typhi and commonly treated by an antimicrobial agent such as cotrimoxazole. On the other hand, herbal usage has risen as an adjunctive therapy to treat many diseases. *Curcuma* (*Curcuma domestica*) is a commonly used herb which consists of curcumin as its major active compound. Curcumin has been known for its antimicrobial effect, but there is no proof regarding the usage of curcumin and cotrimoxazole together. This research was conducted by using typhoid fever model in mice infected by *Salmonella* Typhimurium. Each animal was treated with curcumin, cotrimoxazole, or both. Ileum, spleen, and liver of each animal were isolated and cultured. We found that curcumin-cotrimoxazole combination therapy lowered the antimicrobial effectivity of cotrimoxazole in both intraintestinal and extraintestinal organs. We conclude that curcumin-cotrimoxazole combination therapy in typhoid fever has to be reconsidered.

## 1. Introduction

*Salmonella* Typhi is a Gram-negative bacilli which causes typhoid fever in human. This bacterium may survive in phagosome to escape the immune system. Some complications of typhoid fever are perforation of ileum, bacteremia, and endovascular infection [1, 2]. General therapy of typhoid fever is antimicrobial agents, such as chloramphenicol, ampicillin, and cotrimoxazole (trimethoprim-sulfamethoxazole). Cotrimoxazole is commonly used for typhoid fever therapy in adults and children, including carrier, as an alternative to ampicillin and quinolone. Cotrimoxazole inhibits folic acid synthesis which is needed by bacteria to synthesize nucleic acid [3]. Curcumin is a major active compound of *curcuma* (*Curcuma domestica*), which is widely used as traditional therapy for fever and gastrointestinal problems in Asia. Curcumin has both anti-inflammation and antibacterial effects. It may bind to vitamin D receptor and stimulates expression of an antibacterial protein called cathelicidin [4–6].

Combination between antimicrobial agent and herbal medicine has become a recent trending topic, but there is not enough research to prove that this combination is beneficial to treat typhoid fever. Much more research is needed to find any possible effect of this combination. This study was preceded by an exploration study to ensure antimicrobial effect of both curcumin and cotrimoxazole toward *S. *Typhimurium.

## 2. Materials and Methods

### 2.1. Animal Preparation

Male BALB/c mice underwent acclimatization in laboratory for seven days with free access to meal and water. Animals were randomized into groups and were treated with curcumin, cotrimoxazole, or both. Every mouse was matched to inclusion criteria and did not show any sign of illness prior to infection.

### 2.2. Inoculum Preparation

We used *S. *Typhimurium from Laboratory of Microbiology, Faculty of Medicine, Brawijaya University, Malang, Indonesia. Bacteria were cultured on bismuth sulfite agar and confirmed as *S. *Typhimurium by using MacConkey agar, triple sugar iron IMVIC test, and VITEK2 (bioMerieux). We made inoculum in 10^8^ concentrate. This inoculum was given orally to the animal in 1 cc buffer [7].

### 2.3. Curcumin Preparation

We used curcumin >80% (Sigma-Aldrich). Curcumin was given orally in three different doses for different groups and was emulsified with carboxymethyl cellulose (Sigma-Aldrich) [8]. Therapy with curcumin started three days after infection. We used doses as follows: 100 mg per body weight per day, 150 mg per body weight per day, and 200 mg per body weight per day. Emulsified curcumin was given orally using nasogastric tube for three or five days, according to each therapeutic group.

### 2.4. Cotrimoxazole Preparation

We estimated the animal dose using human-animal conversion factor (0.0026). We used the same dose for all therapeutic groups (1.56 mg per day) divided into two. Cotrimoxazole was given orally using nasogastric tube every twelve hours for three or five days, according to each therapeutic group.

### 2.5. Specimen Culture

According to Ulhaq et al. [9], systemic infection of *S. *Typhimurium begins on the seventh day after initial infection and we confirmed this statement from our exploration study. We cultured distal ileum after three days of therapy. After five days of therapy we cultured distal ileum, spleen, and liver. Each organ was mashed and diluted in buffer. We used bismuth sulfite agar for specimen culture. Every specimen was incubated for twenty-four hours and we counted the black colony using colony counter. Each colony count was converted using the following formula to determine bacterial count inside each specimen [10]:

(1) $$\begin{array}{c} \frac{\left(\text{colony}\;\text{count}\right)\times \left(\text{dilution}\right)\times \left(\text{first}\;\text{dilution}\;\text{concentration}\right)}{\left(\text{specimen}\;\text{weight}\right)}. \end{array}$$

### 2.6. Statistical Analysis

Each data was subjected to statistical analysis. We analyzed our data using paired *t*-test. We considered *P* < 0.05 as significant.

## 3. Results

*Bacteria Colonization in Organs*. Ileum is the part of intestine which has Peyer's patch as main entry of *S. *Typhimurium infection. We found from our exploration study that *S. *Typhimurium has spread via bloodstream into extraintestinal organs such as spleen and liver on the seventh day after infection. We found very different results of curcumin single therapy and curcumin-cotrimoxazole combination therapy.

**Figure 1:**
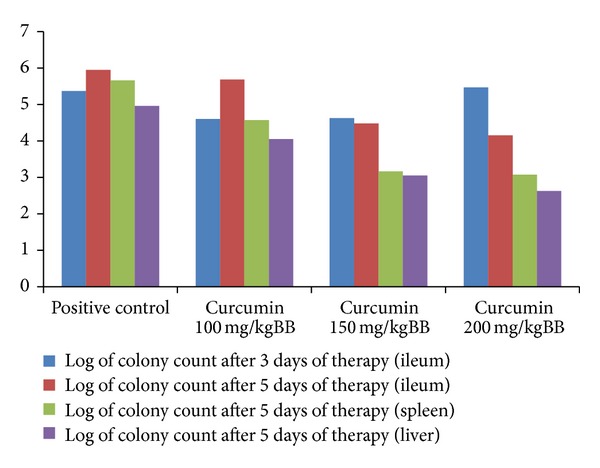
Colony count of ileum, spleen, and liver after curcumin therapy.

According to Figure 1, there was a significant difference between each group of curcumin single therapy. We found an increase of colony count in ileum after three days of therapy. A very different result was obtained from specimen cultures after therapy for five days. Curcumin significantly reduced colonization of bacteria in both intraintestinal and extraintestinal organs after therapy for five days (*P* < 0.05).

**Figure 2:**
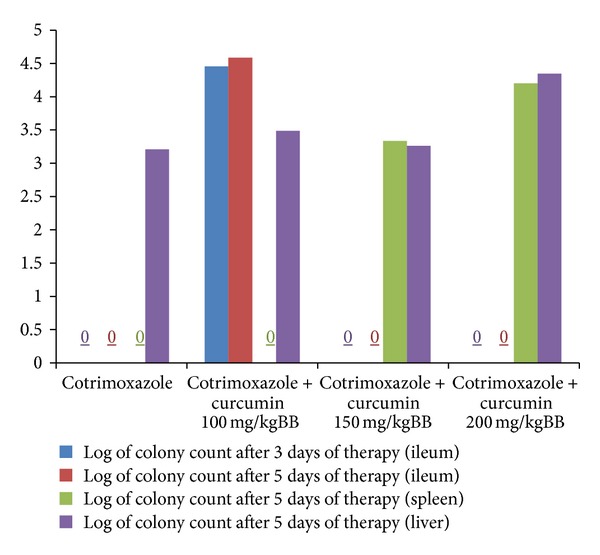
Colony count of ileum, spleen, and liver after curcumin-cotrimoxazole combination therapy.

Figure 2 shows significant difference after curcumin-cotrimoxazole combination therapy compared to curcumin single therapy. After three days using the lowest dose of curcumin (100 mg/kg), we still found bacteria in ileum. Higher dose of curcumin could eliminate these bacteria. Interestingly, after five days of combination therapy, we found that cotrimoxazole could not eliminate extraintestinal bacteria compared to cotrimoxazole single therapy. We found higher colony count when we increased the dose of curcumin in curcumin-cotrimoxazole combination therapy (*P* < 0.05).

After five days of therapy, we found significant difference between colonization in ileum from curcumin single therapy and curcumin-cotrimoxazole combination therapy. We also found significantly different outcomes from curcumin-cotrimoxazole combination therapy after three days and five days, especially in intestine. There was also significant difference in the extraintestinal colonization between curcumin single therapy and curcumin-cotrimoxazole combination therapy for five days (*P* < 0.05).

## 4. Discussions

Following oral ingestion, *S. *Typhimurium undergoes a similar mechanism as *S. *Typhi. The bacteria will colonize gastrointestinal tract, especially ileum, and penetrate into blood vessel to spread systematically [11, 12]. Most bacteria will be killed by gastric acid, so high load of bacteria is needed to reach intestine and manifest clinically [12].

 Curcumin is an active compound which is widely known as anti-inflammatory, anticancerous, and recently, antimicrobial agent. Guo et al. [5] showed that antimicrobial mechanism of curcumin correlates with its ability to bind with vitamin D receptor (VDR) as a potential ligand. This condition promotes expression of cathelicidin antimicrobial peptide (CAMP) and kills the bacteria. Moreover, curcumin may increase mRNA expression of CAMP; thus it may increase cathelicidin level in tissues [5]. Cathelicidin is a small peptide with some structural similarities with other antimicrobial proteins, such as defensin. It has wide spectrum of action toward both Gram-positive and Gram-negative bacteria and also toward some fungi and parasites. Cathelicidin intercalates and infiltrates into bacterial membrane to alter membrane's integrity, but some bacteria are known to have intrinsic resistance toward cathelicidin. These bacteria, such as *Enterococcus faecalis, Streptococcus pyogenes, Salmonella enterica, and Proteus mirabilis,* may synthesize certain proteinase to degrade cathelicidin [13]. Regardless of this, we proved that curcumin still stands a chance against *S. *Typhimurium as an antimicrobial agent.

 Cotrimoxazole is a chemotherapeutic agent which consists of sulfamethoxazole and trimethoprim. Sulfamethoxazole competitively inhibits dihydropteroate enzyme, while trimethoprim inhibits dihydrofolate reductase. Those two substances consecutively inhibit anabolism of folic acid. As an alternative to ampicillin and chloramphenicol, cotrimoxazole is a first line drug for typhoid fever without complication [14].

After three days of therapy using 200 mg/kgBB of curcumin, we found that there was a high load of bacteria that colonized the distal part of ileum. This load decreased after five days of curcumin single therapy. This decrease could not reach zero even with the highest dose of curcumin given in this study. We conclude that curcumin has antimicrobial effect toward *S. *Typhimurium after five days of therapy, and we supposed that lower dose of curcumin might instead promote bacterial growth. Detailed mechanism of this condition still needs further research.

According to Ukil et al. [8] and Jurenka [15], curcumin wields anti-inflammatory effect when given in a dose of at least 50 mg/kg until 100 mg/kg [8, 15], but there was no literature precisely showing its antimicrobial effect in vivo. From this study we found that curcumin cannot be used as a single therapy for treating *S. *Typhimurium infection. But we cannot exclude the possibility that curcumin may show better antimicrobial effect in higher dose, given the pattern of decreased colonization as the dose increases. We could not use a higher dose than 200 mg/kg due to our limited supply of curcumin.

 Curcumin-cotrimoxazole combination therapy showed different effect from curcumin single therapy. Administration of cotrimoxazole together with low dose of curcumin could not kill the bacteria in ileum even after five days of therapy. This condition raised an assumption that low dose of curcumin may alter cotrimoxazole's antimicrobial effect in intestine. Interestingly, this combination therapy also failed to kill extraintestinal colonization of *S. *Typhimurium. We found an increasing number of colonizations in both spleen and liver as we increased the dose of curcumin. We have confirmed effectivity of cotrimoxazole in intraintestinal organs and spleen, although we still found bacterial growth inside liver. Through this finding, we assumed that curcumin may interact with cotrimoxazole and lowers cotrimoxazole's effectivity in killing microbes in extraintestinal organs. 

 A research conducted by Lamont [12] showed that curcumin addition into therapy with ciprofloxacin in infection of *S. *Typhi and *S. *Typhimurium may increase survival rate of the bacteria. Curcumin intervenes the ciprofloxacin's antimicrobial mechanism and alters the environment to become more suitable for bacterial growth. Curcumin increased bacterial proliferation by protecting the bacteria from antimicrobial effect of ciprofloxacin, both in vitro and in vivo via filamentation inhibition and reversed downstream effect of ciprofloxacin [16]. We also found similar effect from combination therapy between patent antimicrobial agent (cotrimoxazole) and curcumin. We found that curcumin lowers the effectivity of antimicrobial effect of cotrimoxazole, but further research is needed to explain the molecular mechanism.

## 5. Conclusion

Curcumin has been known to have antimicrobial properties and to be widely used as adjunctive herbal therapy, especially in Asia. We found that curcumin indeed wields antimicrobial effect toward *S. *Typhimurium, but curcumin lowers antimicrobial effects of cotrimoxazole when these two compounds are given together. According to this, we conclude that combination therapy of curcumin and cotrimoxazole, especially in typhoid fever, needs to be reconsidered.
